# Discovery of SNPs for individual identification by reduced representation sequencing of moose (*Alces alces*)

**DOI:** 10.1371/journal.pone.0197364

**Published:** 2018-05-30

**Authors:** Ida-Maria Blåhed, Helena Königsson, Göran Ericsson, Göran Spong

**Affiliations:** 1 Department of Wildlife, Fish and Environmental Studies, Swedish University of Agricultural Sciences, Umeå, Sweden; 2 Department of Forestry and Environmental Resources, College of Natural Resources, North Carolina State University, Raleigh, North Carolina, United States of America; Universita degli Studi di Sassari, ITALY

## Abstract

Monitoring of wild animal populations is challenging, yet reliable information about population processes is important for both management and conservation efforts. Access to molecular markers, such as SNPs, enables population monitoring through genotyping of various DNA sources. We have developed 96 high quality SNP markers for individual identification of moose (*Alces alces*), an economically and ecologically important top-herbivore in boreal regions. Reduced representation libraries constructed from 34 moose were high-throughput *de novo* sequenced, generating nearly 50 million read pairs. About 50 000 stacks of aligned reads containing one or more SNPs were discovered with the Stacks pipeline. Several quality criteria were applied on the candidate SNPs to find markers informative on the individual level and well representative for the population. An empirical validation by genotyping of sequenced individuals and additional moose, resulted in the selection of a final panel of 86 high quality autosomal SNPs. Additionally, five sex-specific SNPs and five SNPs for sympatric species diagnostics are included in the panel. The genotyping error rate was 0.002 for the total panel and probability of identities were low enough to separate individuals with high confidence. Moreover, the autosomal SNPs were highly informative also for population level analyses. The potential applications of this SNP panel are thus many including investigations of population size, sex ratios, relatedness, reproductive success and population structure. Ideally, SNP-based studies could improve today’s population monitoring and increase our knowledge about moose population dynamics.

## Introduction

The rapid development of sequencing power has greatly facilitated *de novo* genetic studies of wild species [1–3]. Today, high-throughput sequencing and successive development of species-specific molecular markers are often both technically and economically feasible. Single nucleotide polymorphisms (SNPs) as molecular markers have been shown to be reliable, sensitive and highly informative in many species and applications [4–6]. A well-established method to obtain sufficient read depth for accurate SNP calling while reducing the amount of data generated consists in sequencing reduced representation libraries [7, 8]. The DNA is digested with one or more restriction enzymes and the resulting restriction fragments can be size-selected for further optimization prior to sequencing [9]. Reduced representation sequencing has thus proven to be a promising approach for SNP detection in species lacking a reference genome [4, 10].

With low mutation- and error rates, SNPs are ideal for applications that require high confidence in individual genotypes, e.g., individual recaptures and pedigree analyses [11–13]. Additionally, when compared to traditional microsatellite markers, SNPs are better at providing more complete genotypes of higher quality from highly fragmented DNA [14]. Here, fluid-based SNP genotyping in particular has been shown to be particularly amenable to analyses of non-invasive samples of low quality [15]. As SNP genotyping is also cheaper than fragment-based analyses, more loci can be genotyped for the same cost providing better resolution and confidence of individual assignments.

Moose *(Alces alces)*, the largest member of the deer (*Cervidae*) family, is a boreal species with a circumpolar distribution. Through its browsing it influences a wide range of ecosystem processes, such as the composition of woody species and the structure of the tree and shrub layers [16, 17]. Such effects will in turn cascade onto other parts of the ecosystem, for example the insect and bird fauna [18, 19]. In some areas, browsing by moose necessitate adaptive forestry practices and may restrict the range of tree species suitable for rotation forestry [20]. The moose is also an important prey species for large carnivores and a highly valued game species. In Sweden, up to a third of the population is non-randomly harvested every year [21]. The comprehensive and selective harvest strongly affects the population demography (i.e. sex- and age ratios), which in turn affects population dynamics [22–24]. Moose was nearly hunted to extinction in Scandinavia during the 18^th^-19^th^ centuries, the current Swedish population of about 300,000 individuals (pre-harvest) is thus descendent from a much smaller population. The most recent population genetic studies of the Swedish moose suggested restricted gene flow and strong substructuring, with a northern and a southern cluster and a central contact zone [25, 26]. Expanding outside Sweden, Scandinavian moose appear to be genetically separated and show somewhat higher inbreeding rates than the continental European moose [27]. These results motivate population genetic monitoring of the Scandinavian moose to support a sustainable management of the species. To achieve this, knowledge about demographic parameters such as population size and sex ratio is fundamental. Such information could preferably be obtained by recapture studies based on SNP genotyping of non-invasively collected environmental DNA (eDNA) samples. More knowledge about population genetic effects of the resident and migratory behavior of moose would also be of high priority given the opportunity of genotyping individuals with known movement strategies.

The aim of this project was to develop a panel of 96 high quality SNPs for individual identification and sex-determination of moose. We chose a reduced representation sequencing approach to obtain *de novo* genomic sequencing information for autosomal SNP discovery. By specializing the panel for separation of individuals, we target SNPs with relatively high minor allele frequencies (MAF) which risk excluding SNPs informative for population level structure. However, our final panel proved informative at both the individual and population level.

## Materials and methods

### Sampling and location

**Fig 1:**
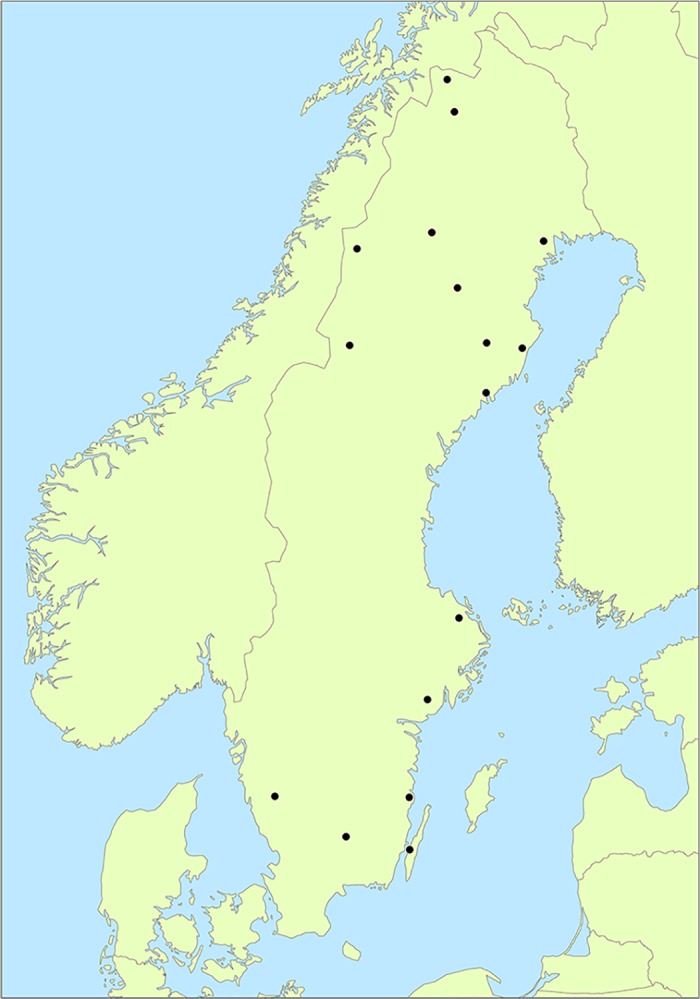
Sampling locations. Moose (n = 34) included for *de novo* sequencing were sampled in the area around 16 locations (1–3 individuals per location) throughout Sweden. The name and geographic coordinates of the sampling locations are provided in S1 Fig.

Thirty-four samples, 16 female and 18 male, were included for reduced representation sequencing and consecutive SNP discovery. The selected individuals were sampled at 16 locations from Abisko (68°N) in the north of Sweden to the island of Öland (56°N) in the south of Sweden (Fig 1). One female with twin offspring were included for biological validation of allele separation. We had access to blood samples collected in connection to telemetry/GPS-collaring of moose during the years 1994–2013 (Permits from the Committee of Ethical evaluation, Umeå: A10/91, A11/91, A12/91, A101/93, A102/93, A103/93, A17/94, A124-06, A116-09, A12-12, A50-12, A205-12, A124-05, A7-03, permits from the committee of Ethical evaluation, Linköping: DNR 77–06). Blood samples were stored at -20°C.

### DNA extraction

Frozen whole blood samples were thawed in 37°C water bath for three minutes during moderate shaking. The sample tubes were kept in motion during the subsequent cooling to room temperature to prevent clotting. Samples were allowed to separate before serum was collected through pipetting of the liquid phase. Extraction of genomic DNA was automatized using the QIAsymphony SP instrument together with the QIAsymphony DNA Mini Kit (Qiagen, Hilden, Germany) following the protocol of the manufacturer. Input sample volume of 200 μl and elution volume 100 μl were found to produce the highest DNA yield. DNA quantity and quality was controlled with spectrophotometry (260:280 ratio, NanoDrop Technologies, Inc.) and gel electrophoresis respectively. We aimed for 5 μg DNA/sample with a DNA concentration of > 50 ng/μl to meet the requirements of our library construction. DNA extracts were stored at -20°C.

### DNA digestion and sequencing

The DNA sequencing followed a reduced representation library approach. The DNA samples were digested with the restriction enzyme Eae I (0.25–1 μl/μg DNA, Takara biotechnology Co., LTD) in 37°C for 18–24 hours. Eae I was chosen based on simulated digestion results from the “RADtag counter”, a resource provided by SciLifeLab Stockholm and originally developed by GenePool, University of Edinburgh. Using the RADtag counter, the number of genomic cut sites and expected average fragment size were calculated using information about GC content (0.45), genome size, and restriction enzyme cut site. The genome size of moose was assumed to be roughly 3 Gb based on known genome sizes of other ungulates i.e. bovine and sheep. Sequencing parameters were based on the algorithm by Altshuler, Pollara [28]. The average fragment size after enzyme digest was estimated to 1500 bp and the chosen size range of fragments for sequencing was 300–500 bp. We aimed for a 20X read depth giving an estimated genomic breadth of coverage of 2%. The MinElute Reaction Cleanup Kit (Qiagen) was used consistent with manufacturer's instructions to purify the samples and to maximize the DNA yield per unit. Size selection of fragments, paired end library construction (2*150 bp) and high throughput multiplex sequencing on one lane of the Illumina HiSeq^TM^ 2500 platform were performed by the National Genomics Infrastructure (NGI), SciLifeLab, Stockholm.

### Data filtering and SNP discovery

The sequence data were delivered demultiplexed. The quality of the reads was evaluated using FastQC 0.10.1 [29] and adapters were removed from the sequences with cutadapt 1.4.2 [30]. Further processing of the sequences was performed using the Stacks 1.13 pipeline [31]. Initially, the feature “process_radtags” was implemented for quality control of the restriction enzyme cut sites and to discard reads with an uncalled base and/or low quality scores. Next, SNPs were called by running “denovo_map.pl” with the parameter settings -m 3, -M 2, -n 1, -t, -H. Subsequent data filtering for detection of high quality SNPs followed the criteria: only one SNP per read, all three allele combinations (XX, XY, and YY) present among the sequenced individuals, data from at least 24 of the 34 individuals and in all sampled locations (14 instead of 16 locations for analysis due to pooling of individuals), minor allele frequency (MAF) > 0.2 and relatively low linkage between SNPs. Flanking regions without variation for a minimum of 40 bp up- and downstream the SNP were required for successful assay (customized primer set) design. Analysis of linkage disequilibrium (LD) and Hardy-Weinberg equilibrium (HWE) were performed with the program Plink 1.07 [32] for targeting independently informative SNPs. The SNP assays were developed by the Fluidigm Corporation, San Fransisco, USA.

### Sex-specific SNPs

For sex-determination of moose, five sex-specific SNPs are included in the SNP panel; three of these markers were developed *de novo*. DNA from six moose individuals were included in the development. Fragments of the *Sry* region were PCR amplified with the primers CerSRYf (5'-TGAACGAAGACGAAAGGTGGCTCT-3') and CerSRYr (5'-TACCCTATTGTGGCCCAGGTTTGT-3') following the method described in Lindsay and Belant [33]. The PCR products were Sanger sequenced using the BigDye Terminator 3.1 kit on a 3730 xl DNA analyzer (Applied Biosystems, Foster City, USA) at the department of Medical Biosciences, Umeå universitet, Sweden. The generated sequences were aligned and a consensus sequence was constructed with BioEdit 7.0.5 (Hall, T. Ibis Therapeutics, Carlsbad, USA) that was manually scanned for sex-specific SNPs. The other two SNP markers for sex-determination: Ce10ay and Ce12ay were previously developed by Nichols and Spong [34]. The sex-specific SNPs were designed to detect the presence of the Y-chromosome (thus failing to provide positive amplification results in females). The minimum threshold for acceptable sex-determination was a positive amplification at three out of five SNPs. Samples amplifying at fewer loci, but not zero, would have been considered ambiguous. The sex-specific SNPs were validated by genotyping of 59 individuals of known sex.

### SNPs for sympatric species diagnostics

Five SNP markers, derived from mitochondrial DNA, are included in the SNP panel for identification and separation of the five different deer species (moose, roe deer (*Capreolus capreolus)*, fallow deer *(Dama dama)*, red deer *(Cervus elaphus)* and reindeer *(Rangifer tarandus*) occurring in the Swedish landscape. This is useful primarily for genotyping of samples of unknown origin including eDNA. DNA from six individuals of each species were included for SNP discovery. The SNPs “Aa_mt_1”, “Aa_mt_4” and “Aa_mt_5” were developed by Sanger sequencing (see details above) of the 12S region with primers developed by Yang, Tan et al. [35]. The SNPs were detected through manual screening following the same procedure as for the sex-specific SNPs described above. The SNPs Ce17mt and Ce19mt were previously discovered by Nichols and Spong [34]. The species diagnostic SNPs were validated through genotyping of all five deer species.

### Validation of autosomal SNPs

The SNP assays were validated by genotyping of Swedish moose DNA of high quality on the Fluidigm Biomark platform (Fluidigm Corporation, San Fransisco, USA). Three “no template controls” (NTCs), i.e. samples containing water instead of DNA, were included. An evaluation was made of the call rate (number of DNA samples successfully genotyped) and performance of the SNP assays regarding assigning samples to a genotype. The later was done visually for each SNP by assessing the clustering of the DNA samples in the Biomark scatter plot plane, aiming for as distinct allele clusters (XX, XY, YY) as possible. SNPs and DNA samples with less than 75% call rate were removed from further analysis. In the assessment of each SNP, DNA samples that were not clearly assigned to a specific genotype were invalidated (i.e. removed from further analysis).

The best performing SNPs (n = 96, all but 10 autosomal) were selected for a final genotyping run of sequenced individuals and additional Swedish moose samples (total n = 59) and three NTCs. The sampled individuals were of known sex (female n = 31, male n = 28). Technical replicates were included (2 samples x 10 replicates, 12 samples x 2 replicates) for calculation of error rates. The number of replicates was chosen based on previous experience from SNP genotyping of high quality DNA in our lab. The design of the replication, including genotyping 2 samples x 10, allowed for discrimination between drop-out and drop-in misprinting errors. The error rate was calculated for each SNP locus as the ratio between the number of mismatching genotypes (among replicates) and the total number of amplified genotypes including both homozygous and heterozygous loci. The presented error rate is the mean value for the SNPs including both groups of replicates (2 x 10 and 12 x 2). Tests of HWE, LD and estimations of F_st_ were performed with Genepop 4.6 [36, 37]. The tests were run with default settings except for that the LD-analysis was based on probability tests. Estimations of heterozygosity, allele frequency and probability of identity (PI) were conducted using GenAlEx 6.502 [38, 39] with default settings. Tests of pairwise relatedness was conducted in GenAlEx and in R 3.4.1. [40] using the program COANCESTRY [41] included in the package “related” 1.0 [42]. To test the SNPs performance as markers for investigations of population structure, we conducted a Bayesian clustering analysis using STRUCTURE 2.3.4 [43] and a multivariate spatial principal component (PCA) analysis. The STRUCTURE analysis was based on an admixture model without prior information about sampling locations. The length of burn-in period was set to 10000 and the number of Markov chain Monte Carlo (MCMC) repetitions after burn-in was 50000. We investigated assumed clusters (*K*) from 1 to 10 with 20 repetitions for each *K*. The most likely number of clusters was selected based on the Δ*K* method, described by Evanno, Regnaut [44]. The PCA analysis was conducted in GenAlEx 6.502 with default settings including a standardized covariance method.

The tests for HWE, LD, expected and observed heterozygosity were performed on a dataset in which individuals from pairs of relatives (*r* > 0.35) were excluded (resulting in n = 50) to avoid biased allele frequencies. Moreover, the genotyping results of the sequenced individuals were controlled against the alleles assigned by the Stacks program to verify that the SNPs were behaving as expected.

## Results

High throughput sequencing of 34 individuals generated nearly 50 million read pairs (2*150 bp) of genomic data. On average, 1.35 million read pairs were produced per sample, varying between 0.61–2.16 million among the samples. The sequences were of satisfying quality with a mean quality score (Illumina 1.9 encoding) exceeding 30 throughout the sequences. More than 240 000 unique stacks of matching sequences were detected using the Stacks “denovo_map” pipeline. Out of these, close to 50 000 stacks contained one or more SNPs. The genomic breadth of coverage after sequencing was estimated to 1.2%.

**Fig 2:**
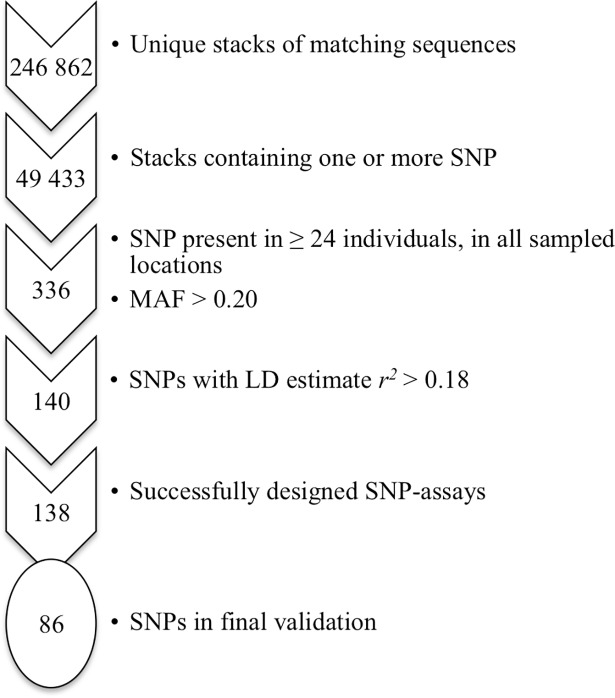
Filtering process for informative SNPs. Schematic overview of the selection process of autosomal SNPs for individual identification, starting at nearly 250 000 stacks of matching DNA sequences and resulting in the selection of 86 SNPs for final validation.

The aim was to develop a panel of 96 SNPs (86 autosomal). Consequently the number of SNPs had to be narrowed down considerably (Fig 2). After applying our criteria for high quality SNPs we ended up with 336 candidates. Finally, the 140 least linked SNPs, i.e. with the lowest *r*^*2*^ score (highest *r*^*2*^ in remaining SNPs: 0.18), were selected for assay design of which 138 passed *in silico* assay design.

### SNP validation and statistics

**Fig 3:**
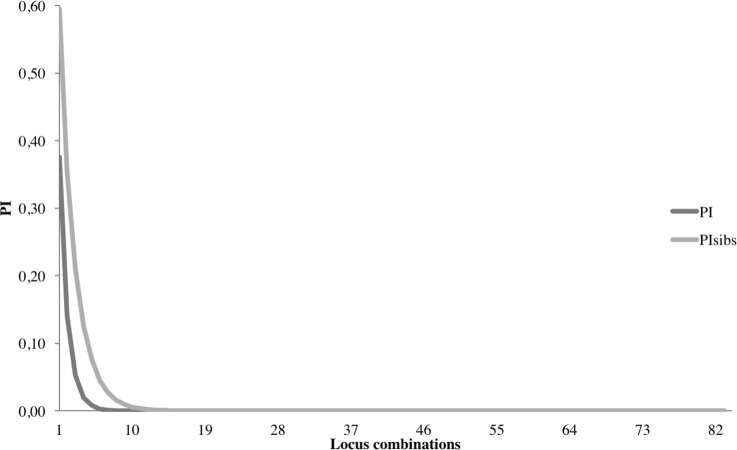
Probability of identity (PI) combining 83 autosomal SNPs. The PI decreases rapidly with increasing number of SNPs and reaches zero (PI < 0.01) with the five most informative SNPs. A combination of 10 SNPs is enough to correctly identify/separate first order relatives (PI_sibs_).

**Fig 4:**
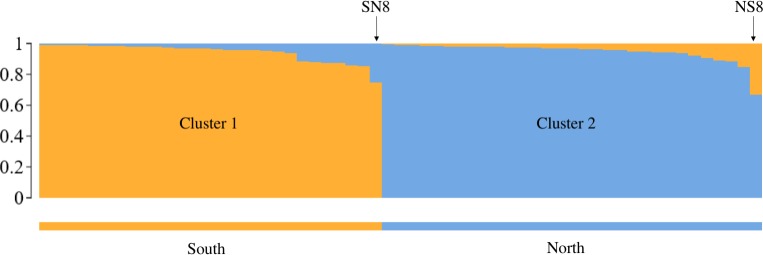
Barplot showing the two clusters suggested by STRUCTURE. The SNP panel separate the 59 moose included in the SNP validation into two genetic clusters. Information about sampling location (south/north) was added to the figure after the analysis to visualize the concordance between assignment of genetic cluster and sampling location. Two individuals, SN8 (south-north 8) and NS8 (north-south 8), are pointed out since they show the most admixture between the two clusters.

The validation by genotyping resulted in the design of a final SNP panel consisting of the 86 best autosomal SNPs. In the final genotyping run, three SNPs did not perform as expected: SNP_12 and SNP_33 whose alleles did not clearly separate and SNP_133 due to low genotyping rate. These three SNPs were removed from further statistical evaluations. Four DNA samples had a genotyping success close or below 75% and were excluded from further assessments due to the increased error rate of such samples. The allelic variants called by the genotyping platform were consistent with the output of the Stacks pipeline except for that the former could detect a somewhat higher number of heterozygotes. The calculated mean error rate per locus for the autosomal SNPs (n = 83) was estimated to 0.001 for the two samples replicated 10 times and 0.003 for the 12 duplicated samples. The mean overall error rate per locus was 0.002. The mean expected and observed heterozygosity of the autosomal SNPs were estimated to 0.48 (*SD* = 0.02) and 0.45 (*SD* = 0.08) respectively. Seven SNPs (SNP_19, SNP_24, SNP_29, SNP_81, SNP_115, SNP_127, SNP_131) deviated from HWE after removing nine individuals from pairs of close relatives (*r* > 0.35). Four SNPs (SNP_19, SNP_29, SNP_115, SNP_152) fell out of HWE including sequenced individuals only. MAF of the SNPs varied from 0.25–0.50 with a mean of 0.43 (S1 Table). Linkage disequilibrium was suggested for 50 out of 3045 SNP pairs (1.5%), at an α-level set at 0.01. The probability that two randomly drawn samples are identified as the same individual approaches zero (PI < 0.01) combining the five most informative SNPs. For first order relatives, 10 SNPs achieve the same resolution (Fig 3). The Lynch and Ritland relatedness estimator [45] gave the highest correlation coefficient between observed and expected values according to simulations in COANCESTRY. The test for pairwise relatednessverified the known relationships among sampled individuals, related coefficient (*r*)): mother-daughter: 0.47, mother-son: 0.35 and brother-sister (assumed twins) 0.51. For validation purposes, Ritland’s relatedness estimator [46] was generated both with COANCESTRY and GenAlEx resulting in consistent values of *r*: 0.46 (mother-daughter), 0.31 (mother-son) and 0.46 (brother-sister). The STRUCTURE analysis resulted in Evanno’s Δ*K* = 2, hence suggesting two genetically differentiated clusters with a north-south separation in accordance with the sampling locations (Fig 4). The individuals (SN8 and NS8) that show the highest admixture between the two clusters where sampled at the locations closest to the contact zone. In the PCA-analysis, the axis explaining most of the variation (10.43%, Eigen value = 35.94) distributed the individuals into a north and a south cluster showing a similar pattern of genetic separation as the STRUCTURE analysis (S2 Fig). The F_st_ value between the northern and the southern clusters was estimated to 0.08, corroborating the genetic separation between these areas.

### Sex-specific SNPs

Four SNP markers for sex-determination passed the validation. They correctly and with high accuracy assigned samples of known sex. Of 28 putative male samples genotyped, all correctly identified as males. Of 31 putative female samples, two identified as males. These two samples have consistently scored as male, leading us to suspect they were originally mislabeled. The apparent error rate for sex-determination thus becomes 0.03, but the likely error rate is zero for samples passing the quality threshold (≥ 75% of loci amplified). The male haplotype for the sex-specific SNPs are given in Table 1. One of the sex-specific SNP markers (Aa_Y_2) did not amplify as expected and was therefore removed from further analyses.

**Table 1:** SNP markers for sex-determination in moose. Five sex-specific SNPs located on the Y-chromosome are included in the panel.

SNP ID	Aa_Y_1*	Aa_Y_2*	Aa_Y_3*	Ce10ay	Ce12ay
Haplotype	T	G	G	T	C

* SNP developed *de novo*

### Species diagnostic SNPs

Five SNPs for separation of sympatric deer species are included in the final 96 marker SNP panel (Table 2). These SNP markers were validated on samples from all five deer species and amplified correctly for all included samples (error rate = 0). One SNP marker (Aa_mt_1) has subsequently been shown, for one roe deer population on the Baltic island Öland, to share the allele of reindeer, making it impossible to separate these two species. However, these populations are not sympatric, as this population of roe deer occur approximately 500 km south of the closest reindeer population. Nevertheless, for applications in regions harboring both roe deer and reindeer, this SNP should ideally be exchanged to avoid uncertainties.

**Table 2:** Species diagnostic SNPs. (a) Five SNP markers are included for separation of five sympatric deer species. (b) The number of expected allele differences between pairs of species.

(a)	Aa_mt_1*	Aa_mt_4*	Aa_mt_5*	Ce17mt	Ce19mt
Moose	C	C	C	T	G
Roe deer	A/C	T	C	T	A
Red deer	C	T	T	T	A
Fallow deer	C	T	T	C	G
Reindeer	C	T	C	T	A
(b)	Moose	Roe deer	Red deer	Fallow deer	Reindeer
Moose	-	-	-	-	-
Roe deer	2–3	-	-	-	-
Red deer	3	1–2	-	-	-
Fallow deer	3	3–4	2	-	-
Reindeer	2	0–1	1	3	-

* SNP developed *de novo*.

The sequence information of the SNP-assays is available from the Zenodo repository: https://doi.org/10.5281/zenodo.1237474.

## Discussion

Our final panel of 96 SNPs based on *de novo* reduced representation sequencing, including markers for both sex-determination and sympatric deer species diagnostics, is a suitable tool for moose individual identification and population monitoring which can ensure important information for wildlife monitoring and management.

Out of 50 million read pairs of data, more than 50 000 putative SNPs were detected. Hence, sequencing produced a sufficient number of high quality reads for successful SNP discovery. Sequencing was performed in multiple runs and since enough SNPs were detected in the first data delivery, we did not include data from subsequent sequencing efforts. As an effect, the lower than expected amount of data per sample resulted in a lower than expected read depth per sample. This could have made detection of high quality SNPs difficult with an increased risk of including spurious alleles arising from sequencing errors. Due to variations in the number of reads produced from each sample and to avoid excluding informative SNPs sequenced at low depth at the individual level, the minimum stack depth per individual was set at a modest value of three. However, the stack depth per individual should be multiplied with the total number of individuals that the SNP was detected in (i.e. 24–34), leading to stack depth of 72–102 X. Hence, the SNPs passing the quality criteria are true genomic variations and since nearly all of the SNPs have a MAF above 0.3, the risk of erroneous alleles at the population level is practically absent. Moreover, the validation by assay-based genotyping confirmed the selected SNPs to be polymorphic.

The genotyping error rate for the final SNP panel was low which is in line with our expectations. Three of the final autosomal SNPs did not meet the quality requirements in the validation genotyping run. Our experience is that out of 96 SNPs, a few can fail due to different reasons (e.g., evaporation, primer-dimer binding during PCR etc.). Consequently, these three SNPs are likely to perform well in coming genotyping efforts.

In the validation of the autosomal SNPs, seven markers were found to deviate from HWE. Deviations from HWE are expected to some degree when studying natural animal populations since the assumptions of the Hardy-Weinberg model are rarely met [47]. To exemplify this in the moose, the competitive reproductive behavior as well as the migratory behavior of males violate the assumptions of random mating and closed populations. Signals of linkage disequilibrium were detected in 1.5% of the possible pairwise SNP combinations in the final validation. We do not know if these are true cases of linkage or simply effects of demographic processes in the past. Efforts to present whole genome data from moose will resolve this question in the near future.

The statistical power of these SNPs to resolve individuals is high (Fig 3), their error rate is at 0.2% and they follow the expected inheritance patterns in our kinship triad (mother with twin offspring) with one apparent exception. The *r* value between the siblings was close to 0.5, as was the value between the mother and the female calf. But the *r* value between mother and the male calf was surprisingly low (0.35). Stochastical variation in the *r* value estimate is unlikely to generate such a low value and the low error rates of the SNP panel suggest that this deviation might have its explanation in factors other than the SNP panel's performance. As with the two female samples unexcitingly displaying as males, mislabeling of samples somewhere in the handling process could be a possible explanation also in this case. To have assumed kinship patterns upset or detect errors are not uncommon when adding genetic information to existing data sets.

The north-south genetic differentiation detected by the SNP panel, as shown by results from STRUCTURE-, PCA- and F_st_ analyses, is in line with previous studies from the same area [25, 26, 48]. The level of substructuring that can be detected with our SNPs is yet to be determined, but in combination with individual-based analyses (i.e. pedigree analyses) the panel looks like a promising tool for population monitoring. The lack of sequencing information from individuals across the entire geographic area, could cause an ascertainment bias. However, this is unlikely to affect our detection of SNPs, as the central population is known to contain an admixture of genotypes between the northern and southern parts [25]. The geographic application range of the SNP panel is still uninvestigated. Based on the postglacial colonization patterns of moose, the SNPs are potentially polymorphic across Scandinavia. Moving further east, the moose is increasingly genetically differentiated [27], hence the levels of polymorphism of our markers are at present unknown.

The possibility of sex-determination facilitates pedigree construction and enables estimations of sex ratios, which is important information for e.g., wildlife management. One of the five sex-specific SNPs failed to amplify during the validation but worked in later genotyping runs. That amplification fails is always a risk and emphasizes the importance of running enough markers and/or to include replicates. Though the sex of all individuals in the SNP validation was correctly determined, it should be mentioned that assigning females based on the absence of a reaction, as we do here, comes with less certainty than having a positive confirmation. This is since a male sample that fails in the genotyping will appear as a female. The solution to this potential caveat is to use a sample quality threshold and enough markers to reliably determine the sex.

Our primary goal was to select SNPs with high MAF and wide geographical distribution of alleles in order to optimize the panel for individual identification of moose. This goal was achieved by the development of a panel of SNPs with high confidence in assigning individuals. Our requirement that alleles show a good geographical distribution comes at the risk of losing power to detect population structure. Despite this, the autosomal SNPs also do well in detecting population substructuring based on allele frequency differences. The final SNP panel is thus promising for studies of moose both at the individual and population level.

## Supporting information

S1 Fig Geographical coordinates of sampling locations. Moose (n = 34) included for *de novo* sequencing were sampled in the area around 16 locations throughout Sweden.
(PDF)

S2 Fig PCA plot showing the two top principal components. Principal component 1 (PC 1), explaining 10.43% of the variation (Eigen value = 35.94), separates the 59 samples into a northern- and a southern cluster in accordance with the sampling locations (South/North). PC 2 explains 5.28% of the variation (Eigen value = 18.20).
(PDF)

S1 Table SNP allelic variants and population genetic statistics. (PDF)
