# Supplementary material for: Discovery of SNPs for individual identification by reduced representation sequencing of moose (Alces alces)
Source: PLoS One. 2018 May 30;13(5):e0197364. doi: 10.1371/journal.pone.0197364 (PMC5976195; doi:10.1371/journal.pone.0197364)

Nr	Location	°N	°E
1	Abisko	68,347970	18,825928
2	Nikkaluokta	67,852211	19,006326
3	Arjeplog	66,051578	17,892784
4	Hemavan	65,815355	15,086450
5	Ängesbyn	65,773449	22,015888
6	Malå	65,184875	18,742021
7	Bågede	64,343931	14,809501
8	Hällnäs	64,311348	19,624699
9	Robertsfors	64,191945	20,849055
10	Nordmaling	63,569826	19,498922
11	Gimo	60,172070	18,179993
12	Östermalma	58,951340	17,161734
13	Mark	57,474426	12,749501
14	Misterhult	57,462658	16,543984
15	Växjö	56,879143	14,771973
16	Öland	56,681653	16,522033

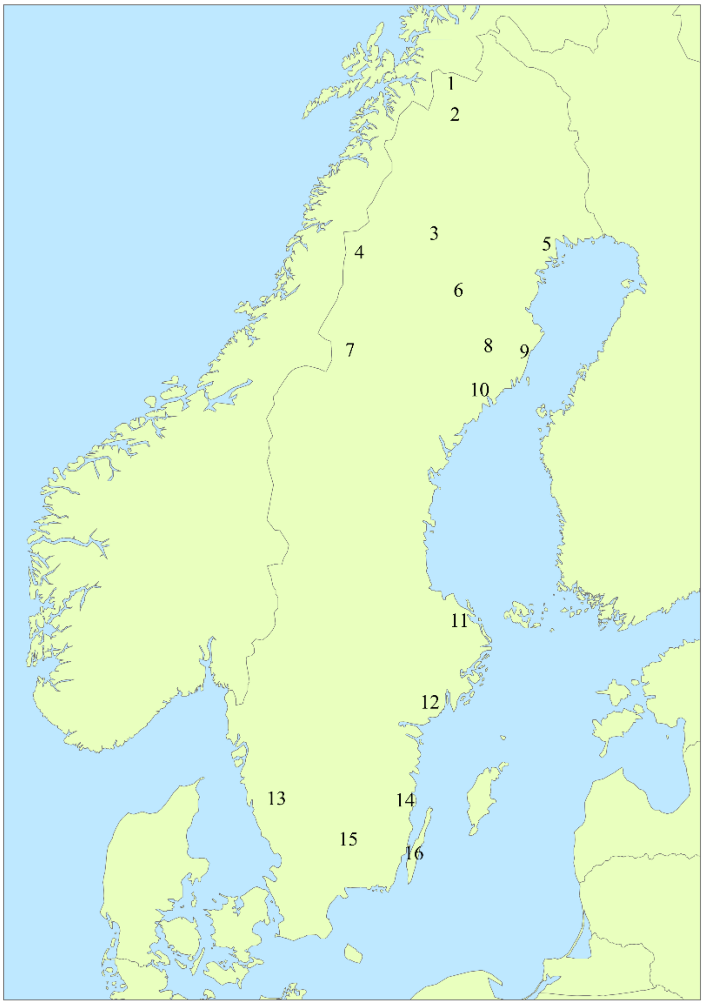

Supplement: S1 Fig — Moose (n = 34) included for de novo sequencing were sampled in the area around 16 locations throughout Sweden. (PDF) [file pone.0197364.s001.pdf]
