# Supplementary figures and images for: Discovery of SNPs for individual identification by reduced representation sequencing of moose (Alces alces)
Source: PLoS One. 2018 May 30;13(5):e0197364. doi: 10.1371/journal.pone.0197364 (PMC5976195; doi:10.1371/journal.pone.0197364)

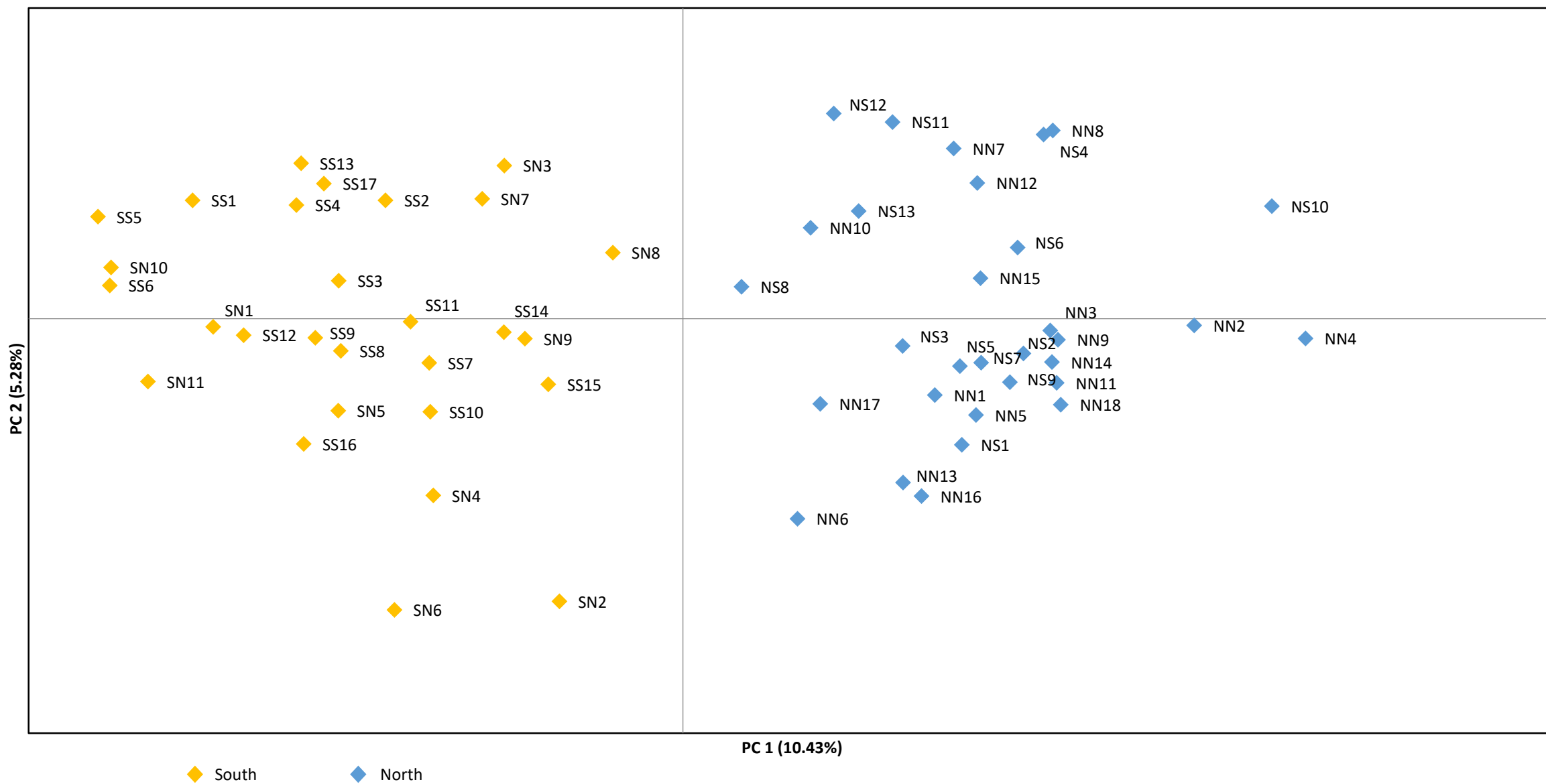

Supplement: S2 Fig — Principal component 1 (PC 1), explaining 10.43% of the variation (Eigen value = 35.94), separates the 59 samples into a northern- and a southern cluster in accordance with the sampling locations (South/North). PC 2 explains 5.28% of the variation (Eigen value = 18.20). (PDF) [file pone.0197364.s002.pdf]
