# Supplementary material for: Discovery of SNPs for individual identification by reduced representation sequencing of moose (Alces alces)
Source: PLoS One. 2018 May 30;13(5):e0197364. doi: 10.1371/journal.pone.0197364 (PMC5976195; doi:10.1371/journal.pone.0197364)
Supplement: S1 Table — (PDF) [file pone.0197364.s003.pdf]

SNP ID	Allele	MAF	H obs	H exp	HWE (*p*)
**Autosomal SNPs**					
**Aa_SNP_1**	T/C	0,475	0,540	0,500	0,778
**Aa_SNP_4**	G/T	0,475	0,520	0,480	0,766
**Aa_SNP_5**	A/C	0,500	0,480	0,493	1,000
**Aa_SNP_7**	A/G	0,466	0,520	0,499	1,000
**Aa_SNP_10**	G/A	0,373	0,531	0,490	0,773
**Aa_SNP_12**	G/A				
**Aa_SNP_15**	G/A	0,474	0,480	0,487	1,000
**Aa_SNP_16**	T/C	0,492	0,460	0,498	0,577
**Aa_SNP_17**	G/C	0,397	0,400	0,493	0,249
**Aa_SNP_19**	A/C	0,381	0,327	0,498	0,020
**Aa_SNP_23**	T/C	0,465	0,388	0,500	0,157
**Aa_SNP_24**	A/C	0,360	0,320	0,497	0,012
**Aa_SNP_26**	C/G	0,448	0,469	0,500	0,774
**Aa_SNP_28**	C/T	0,475	0,460	0,484	0,771
**Aa_SNP_29**	T/C	0,415	0,200	0,487	0,000
**Aa_SNP_30**	A/G	0,458	0,480	0,499	0,782
**Aa_SNP_31**	A/G	0,466	0,500	0,466	0,770
**Aa_SNP_33**	C/T				
**Aa_SNP_35**	G/A	0,449	0,354	0,495	0,075
**Aa_SNP_36**	C/T	0,373	0,510	0,493	1,000
**Aa_SNP_38**	A/C	0,483	0,560	0,499	0,570
**Aa_SNP_45**	T/A	0,331	0,388	0,479	0,227
**Aa_SNP_46**	G/A	0,339	0,460	0,498	0,579
**Aa_SNP_47**	T/C	0,339	0,520	0,487	0,776
**Aa_SNP_48**	A/T	0,415	0,500	0,500	1,000
**Aa_SNP_52**	C/T	0,441	0,580	0,484	0,249
**Aa_SNP_53**	A/C	0,431	0,340	0,442	0,116
**Aa_SNP_54**	T/C	0,356	0,420	0,495	0,262
**Aa_SNP_55**	A/G	0,466	0,383	0,494	0,142
**Aa_SNP_56**	A/T	0,424	0,542	0,492	0,572
**Aa_SNP_57**	C/T	0,449	0,490	0,475	1,000
**Aa_SNP_58**	G/A	0,483	0,426	0,498	0,381
**Aa_SNP_59**	T/C	0,373	0,560	0,471	0,241
**Aa_SNP_60**	G/T	0,390	0,380	0,442	0,344
**Aa_SNP_62**	C/A	0,475	0,542	0,486	0,556
**Aa_SNP_63**	T/C	0,368	0,438	0,474	0,554
**Aa_SNP_65**	A/T	0,432	0,469	0,497	0,773
**Aa_SNP_66**	A/G	0,474	0,440	0,497	0,403
**Aa_SNP_67**	C/T	0,475	0,400	0,487	0,250
**Aa_SNP_68**	A/G	0,446	0,510	0,497	1,000
**Aa_SNP_69**	T/C	0,456	0,400	0,435	0,531
**Aa_SNP_73**	T/C	0,377	0,540	0,484	0,560

**Aa_SNP_74**	A/G	0,305	0,440	0,420	1,000
**Aa_SNP_75**	T/C	0,441	0,460	0,500	0,584
**Aa_SNP_76**	T/C	0,432	0,400	0,471	0,363
**Aa_SNP_79**	G/T	0,475	0,460	0,484	0,773
**Aa_SNP_81**	C/T	0,388	0,340	0,498	0,024
**Aa_SNP_82**	C/T	0,407	0,531	0,475	0,554
**Aa_SNP_84**	T/C	0,439	0,480	0,487	1,000
**Aa_SNP_90**	G/C	0,421	0,380	0,442	0,340
**Aa_SNP_92**	G/A	0,384	0,380	0,455	0,234
**Aa_SNP_93**	G/A	0,415	0,480	0,480	1,000
**Aa_SNP_94**	T/C	0,466	0,480	0,487	1,000
**Aa_SNP_95**	A/C	0,466	0,440	0,497	0,413
**Aa_SNP_97**	C/T	0,362	0,460	0,500	0,577
**Aa_SNP_98**	C/T	0,500	0,440	0,480	0,554
**Aa_SNP_100**	A/G	0,415	0,460	0,484	0,772
**Aa_SNP_102**	G/A	0,415	0,460	0,442	1,000
**Aa_SNP_106**	T/A	0,446	0,600	0,497	0,255
**Aa_SNP_107**	T/C	0,458	0,440	0,497	0,402
**Aa_SNP_108**	T/C	0,371	0,440	0,497	0,410
**Aa_SNP_110**	C/T	0,466	0,480	0,497	0,782
**Aa_SNP_112**	C/A	0,407	0,438	0,500	0,399
**Aa_SNP_113**	G/A	0,483	0,531	0,498	0,779
**Aa_SNP_115**	G/T	0,373	0,306	0,493	0,010
**Aa_SNP_116**	C/T	0,466	0,417	0,444	0,745
**Aa_SNP_119**	G/A	0,314	0,380	0,412	0,731
**Aa_SNP_125**	C/T	0,448	0,500	0,498	1,000
**Aa_SNP_126**	A/T	0,458	0,480	0,449	0,756
**Aa_SNP_127**	A/G	0,457	0,340	0,500	0,026
**Aa_SNP_128**	T/C	0,441	0,500	0,498	1,000
**Aa_SNP_129**	G/T	0,424	0,480	0,497	0,780
**Aa_SNP_131**	T/G	0,331	0,320	0,499	0,012
**Aa_SNP_132**	T/C	0,492	0,520	0,497	1,000
**Aa_SNP_133**	G/T				
**Aa_SNP_134**	C/T	0,491	0,612	0,495	0,152
**Aa_SNP_135**	C/T	0,456	0,520	0,471	0,562
**Aa_SNP_137**	C/G	0,415	0,580	0,466	0,134
**Aa_SNP_138**	T/C	0,254	0,400	0,493	0,259
**Aa_SNP_139**	T/A	0,347	0,388	0,459	0,351
**Aa_SNP_140**	A/T	0,475	0,490	0,490	1,000
**Aa_SNP_150**	A/G	0,381	0,300	0,394	0,147
**Aa_SNP_151**	C/T	0,415	0,460	0,466	1,000
**Aa_SNP_152**	A/G	0,483	0,583	0,486	0,241
**Aa_SNP_153**	G/T	0,455	0,440	0,497	0,403
**Aa_SNP_154**	A/G	0,500	0,420	0,498	0,268

**Sex-specific SNPs**	
**Aa_Y_1**	T/-
**Aa_Y_2**	G/-
**Aa_Y_3**	G/-
**Ce10ay**	T/-
**Ce12ay**	C/-
**Species diagnostic SNPs**	
**Aa_mt_1**	C/-
**Aa_mt_4**	C/-
**Aa_mt_5**	C/-
**Ce17mt**	T/-
**Ce19mt**	G/-
